# On improving the sustainability of peer review

**DOI:** 10.1371/journal.pbio.3003127

**Published:** 2025-03-25

**Authors:** Daniel Routledge, Nonia Pariente, On Behalf of the PLOS Biology Staff Editors

**Affiliations:** 1 Public Library of Science, San Francisco, California, United States of America

## Abstract

The term “reviewer fatigue” has become only too familiar in scientific publishing. This editorial discusses how we can ease the burden on reviewers to make the peer review system more sustainable, while streamlining the publication process for authors.

## Teaser

The term “reviewer fatigue” has become only too familiar in scientific publishing. How can we ease the burden on reviewers to make the peer review system more sustainable, while streamlining the publication process for authors?

Scientists frequently lament that the current system for scientific peer review is “broken”, with the number of invitations to review far outweighing the capacity to complete them, resulting in decreased reviewer acceptance rates, increased numbers of overdue or never delivered reports, and increased times to decision [1–4]. *In lieu* of an alternative system, which is a whole other discussion in itself, how can we ease the burden on reviewers to make the one that we have more sustainable?

Whether you are a group leader struggling to find the time to complete a review, an editor having difficulty securing sufficient reviewers, or a researcher waiting months to receive an editorial decision, the headache that peer review can be is felt across all aspects of scientific publishing. And this is to say that there is no blame to place on individuals; the growth of scientific research and the concomitant increase in the number of publications over the past decades [2,5], alongside the ‘publish or perish’ culture to produce more publications more frequently [6], has amplified the demand for expert reviewers. The term “reviewer fatigue” has therefore sadly become quite familiar. Ironically, you may have found yourself in a position where the reviewer reports for your own paper are overdue, while the report(s) that you agreed to write also tick over past their deadline. But despite its flaws, it is also widely agreed that peer review is valuable (nay, integral) to science, and helps improve the quality and rigor of published articles [2]. So, it appears we have reached an impasse - where do we go from here?

A clear starting point is trying to make the system more efficient and streamlining the peer review process. At *PLOS Biology*, we actively encourage portable peer review (PPR), where we will consider manuscripts on the basis of reviews received at journals from other publishers. If the work meets editorial criteria for further consideration, the editors will contact the previous journal to confirm the previous history, share the reviews and, if possible, original reviewer identities. Staff editors work with our Academic Editors to arbitrate these reports directly, avoiding or minimizing further peer-review and thereby streamlining the process for authors, as well as reducing the burden on reviewers. We have published many articles in this way, with considerably shorter times to publication. However, this requires journals to be open to sharing reports and reviewer identities with other publishers. A system that takes into account existing reviews reduces futile cycles of peer review and is advantageous to both authors and reviewers, so we call on all publishers to agree to sharing reports and reviewers’ identities (with consent) with each other.

Another initiative that we are part of, to simplify the process of manuscript assessment by peers, is Review Commons, a platform that organizes review of preprint manuscripts before they are submitted to a journal. The Refereed Preprint (the manuscript along with its reviewer reports and the authors’ response), is transferred on the authors’ behalf to bioRxiv and can be submitted to any of the 28 affiliate journals, and easily transferred between up to three journals in the case of a rejection. We are also a Peer Community In friendly journal, and will work with existing reviewer reports when we receive articles peer-reviewed in any of the PCIs. Similarly to PPR, these initiatives streamline the process by omitting the need for serial re-review, with the added benefit of journal-agnostic peer review, allowing reviewers to assess a study on its merits, without any potential bias from knowing where it was submitted.

Okay, so there are ways the process can be streamlined, but what about the time it actually takes to review? In a world where scientists are overworked and struggle to find the time to complete their reviews, how could we incentivize participation in peer review and submission of reports on time? About 85% of researchers consulted consider that institutions should require and, importantly, recognize peer review contributions [2]. This is an important concept; reviewers help advance science, and this work should be adequately valued, in the same way that other factors (e.g., publications and technical expertise) are valued in funding and hiring. Another issue that has been discussed is paying peer reviewers [7]. However, several concerns have meant this idea is unlikely to garner widespread support, including (but not limited to) diminishing the quality of reports, misguided motivation (i.e., for monetary gain), and the fear that publishers would increase their prices to finance such an initiative [8,9].

More controversially, artificial intelligence (AI) has crept its way into the conversation. It has been suggested that AI could be used to assist reviewers by assessing and summarizing papers, consolidating notes, and even compiling reviewer reports, saving reviewers both time and effort [10,11]. However, even in a world where AI is advanced enough to accurately summarize a scientific article, peer review is much more than just summarizing; it is critiquing and contextualizing the paper, requiring unique expertise to evaluate the data or methodology from another perspective and make insightful recommendations. In addition, there are numerous other issues surrounding confidentiality and data protection (e.g., when uploading manuscripts to online generative AI tools), and regulation of its use. At *PLOS Biology*, we do not permit the use of AI to review a paper, only to polish the text of a review that has already been written (which may be particularly beneficial for non-native English speakers), and this must be declared to the editors and authors (see our policies here). Limitations of AI aside, the concern here is that scientists feel the need to find such corner-cutting methods, highlighting the degree to which fatigue has set in.

Rather, we should be advocating for more sustainable changes to the peer review process that allow for effective, quality peer review without resulting in burnout. Another way this could be achieved is to expand the reviewer pool, thus spreading the workload further, and reducing regional bias. From 2013–2017, ‘established regions’ (such as the USA and UK) reviewed more than ‘emerging regions’ (such as China and India) relative to their respective article outputs [2]. Approaching more scientists from underrepresented and ‘emerging’ regions, many of which are happy to participate in the process of peer review, would democratize the process and help alleviate excessive demands on a small pool of people. A more consistent use of ORCiD identifiers would be beneficial in this regard, as it would aid editors in identifying suitable reviewers and disambiguating individuals with the same name when doing conflict of interest checks, for example. Postdoctoral researchers are also a possible source of suitable experts, quite often overlooked. As well as advocating for editors to invite more postdocs, we also encourage group leaders who do not have the time to review a paper themselves, to recommend postdocs with appropriate expertise and to always declare when they co-review with members of their labs, who may be approached directly in the future.

Finally, another consideration is whether the timelines of peer review are excessively demanding. The median time to complete a reviewer report is around 16 days [2] – and yet journal deadlines are often shorter than this. Journal deadlines should probably be adjusted to be more realistic, unless exceptional circumstances warrant an expedited process – at *PLOS Biology*, we ask that reviews be submitted within 2 weeks, and are happy to provide short extensions, within reason. In addition, with the option of preprinting your manuscript as a means to make studies immediately available to the community, for some, time-to-publication may not be as significant a factor as it used to be. Would you mind if your paper took slightly longer to publish if it meant relieving some of the stress and pressure on reviewers?

In addition to broadening the reviewer pool, we hope that initiatives like PPR, Review Commons and PCI, which help reduce serial rounds of review, will be widely adopted and thus streamline the process for authors and lessen the burden on reviewers. Equally, given the increasing appetite for overhauling the current system altogether [9], we should remain open-minded to experimentation with new peer review structures and initiatives; after all, scientists are problem-solvers. If the solution was easy, wouldn’t we have figured it out by now?
